# Key Aspects in Nutritional Management of COVID-19 Patients

**DOI:** 10.3390/jcm9082589

**Published:** 2020-08-10

**Authors:** Alfredo Fernández-Quintela, Iñaki Milton-Laskibar, Jenifer Trepiana, Saioa Gómez-Zorita, Naroa Kajarabille, Asier Léniz, Marcela González, María P. Portillo

**Affiliations:** 1 Nutrition and Obesity Group, Department of Pharmacy and Food Science, University of the Basque Country (UPV/EHU) and Lucio Lascaray Research Institute, 01006 Vitoria, Spain; alfredo.fernandez@ehu.eus (A.F.-Q.); inaki.milton@ehu.eus (I.M.-L.); naroa.kajarabille@ehu.eus (N.K.); asier.leniz@gmail.com (A.L.); mariapuy.portillo@ehu.eus (M.P.P.); 2 CIBEROBN Physiopathology of Obesity and Nutrition, Institute of Health Carlos III, 01006 Vitoria, Spain; 3 Bioaraba Health Research Institute, 01009 Vitoria, Spain; 4 Basque Health Service (Osakidetza), Integrated Health Care Organization Araba, 01009 Álava, Spain; 5 Nutrition and Food Science Department, Faculty of Biochemistry and Biological Sciences, National University of Litoral and National Scientific and Technical Research Council (CONICET), Santa Fe 3000, Argentina; maidagon@fbcb.unl.edu.ar

**Keywords:** COVID-19, SARS-CoV-2, nutrition, malnutrition, nutritional support, bioactive compounds

## Abstract

This review deals with the relationship among nutrition, the immune system, and coronavirus disease 2019 (COVID-19). The influence of nutrients and bioactive molecules present in foodstuffs on immune system activity, the influence of COVID-19 on the nutritional status of the patients, and the dietary recommendations for hospitalized patients are addressed. Deficient nutritional status is probably due to anorexia, nausea, vomiting, diarrhea, hypoalbuminemia, hypermetabolism, and excessive nitrogen loss. There is limited knowledge regarding the nutritional support during hospital stay of COVID-19 patients. However, nutritional therapy appears as first-line treatment and should be implemented into standard practice. Optimal intake of all nutrients, mainly those playing crucial roles in immune system, should be assured through a diverse and well-balanced diet. Nevertheless, in order to reduce the risk and consequences of infections, the intakes for some micronutrients may exceed the recommended dietary allowances since infections and other stressors can reduce micronutrient status. In the case of critically ill patients, recently published guidelines are available for their nutritional management. Further, several natural bioactive compounds interact with the angiotensin-converting enzyme 2 (ACE2) receptor, the gateway for severe acute respiratory syndrome (SARS) and severe acute respiratory syndrome coronavirus 2 (SARS-CoV-2). Natural bioactive compounds can also reduce the inflammatory response induced by SARS-CoV-2. These compounds are potential beneficial tools in the nutritional management of COVID-19 patients.

## 1. Introduction

In December 2019, there was an outbreak of pneumonia of unknown cause in Wuhan, Hubei Province, China, which affected more than 60 people on the twentieth of that month. On 31 December, the Wuhan Municipal Health Committee informed the World Health Organization (WHO) that 27 people had been diagnosed with pneumonia of unknown cause, being 7 of them critically ill [1]. By January, the first cases of coronavirus disease 2019 (COVID-19) had been reported outside China: two in Thailand and one in Japan. Then, the rapid spread of the disease prompted the WHO to declare it as a health emergency of international concern, based on the impact the virus could have on underdeveloped countries with fewer health infrastructure. By that date, the disease had been detected in all provinces of mainland China, and cases were also diagnosed in 15 other countries. In March, the disease was already in more than 100 territories worldwide, and recognized as a pandemic by the WHO. At present, the number of confirmed cases continues to grow.

**Figure 1:**
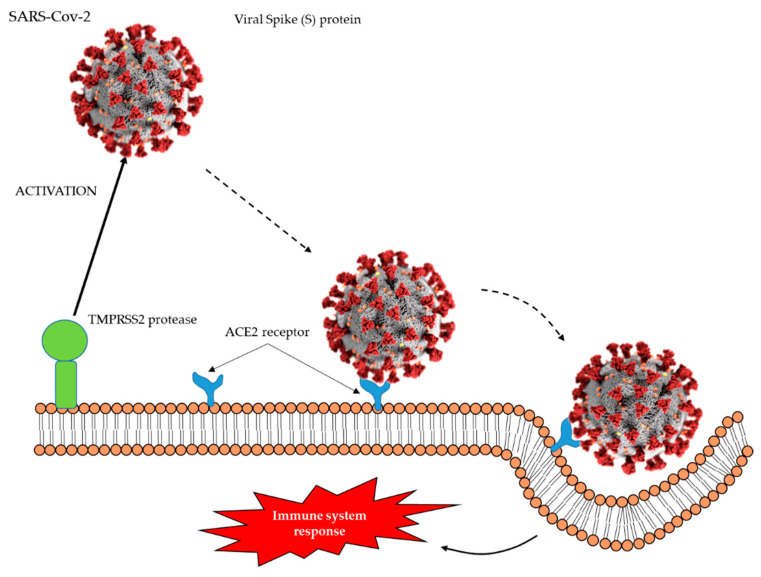
Severe acute respiratory syndrome coronavirus 2 (SARS-CoV-2) virus uses the angiotensin-converting enzyme 2 (ACE2) receptor to enter the host cell and the transmembrane protease serine 2 (TMPRSS2) for Spike (S) protein priming.

The virus that produced COVID-19 is the severe acute respiratory syndrome coronavirus 2 (SARS-CoV-2), an enveloped positive-sense RNA virus that mainly affects the respiratory system, being the spread of droplets generated by an infected subject the main route of transmission. When SARS-CoV-2 binds angiotensin-converting enzyme 2 (ACE2) receptors in the alveolar epithelial cells, the immune system responds through inflammation-related manifestation as well as antigen-presenting cell recruitment (Figure 1). The disease can be asymptomatic or present mild affection of the upper respiratory tract, while in the most severe cases is characterized by acute respiratory distress syndrome, heart failure, and septic shock [2]. Moreover, as the disease advances, multi-organ failure has also been reported as the result of uncontrolled acute inflammation. Indeed, the immune response against the virus triggered by this uncontrolled inflammation results in pulmonary tissue damage, which in turn reduces lung capacity. The tissue damage produced by SARS-CoV-2 at the alveolar level is characterized by pathological changes of the tissue, infiltration, and hyperplasia. Besides respiratory failure, other features have also been described as common in critically ill patients of COVID-19, among them infiltration of immune cells into lung injuries, high levels of inflammatory response, thrombosis, and multi-organ failure [3].

Additionally, the presence of other chronic diseases in the patient may exacerbate the inflammatory response derived from COVID-19, increasing the risk for adverse effects and mortality. In this regard, the systemic inflammation present in subjects with non-communicable diseases (NCDs), such as diabetes, tend to make the respiratory symptoms of the infection more severe [4]. On the other hand, it has been reported that excessive body mass index (BMI) and/or adiposity are considered risk factors for complications derived from COVID-19 infection, especially in patients with compromised heart and lung function [5]. Similarly, the damage in blood vessels that commonly exist in patients with diabetes and/or high-blood pressure increases the risk of these subjects to suffer COVID-19 derived thrombotic complications.

Due to the novelty of this pandemic, scientific community is currently looking for effective vaccines, as well as drugs to treat the pathology. One of the biggest challenges is focused on reducing inflammation, without compromising the correct immune response of the patient. In this scenario, science should focus not only in effective drugs but also in nutrition. The importance of an adequate nutritional status and dietary habits has been extensively highlighted in the COVID-19 pandemic, not only as a matter of avoiding the presence of NCDs that can result in more severe infections, but also as a way to modulate the inflammatory status of the patients. Indeed, the underestimation of the importance of nutrition in COVID-19 patients can dramatically affect the outcome of these patients [3,6].

The aim of the present review is to analyze the current knowledge on the relationship among nutrition, the immune system, and COVID-19 in order to formulate clinical advice and to highlight the directions for future research. Thus, the influence of nutrients and bioactive molecules present in foodstuffs on immune system activity, the influence of COVID-19 on nutritional status of patients and the dietary recommendations for COVID-19 hospitalized patients are addressed in this manuscript.

## 2. The Role of Specific Dietary Nutrients on Immune System Function and COVID-19 Disease

The influence of nutrition in the immune system has been widely reported. In addition, recent studies have also highlighted the influence of both, an adequate nutritional status and the appropriate intake of specific nutrients in COVID-19. Nevertheless, due to the novelty of the disease, information regarding the effects of some nutrients is scarce, and in some cases, this information comes from ecological studies. Therefore, it seems plausible that part of the information included in this section may be updated in the upcoming months as the result of the research that is currently ongoing. Protein deficiency is linked to impaired immune system function, mainly due to its negative effects on both, the amount of functional immunoglobulins and gut-associated lymphoid tissue (GALT). Besides quantity, the quality of proteins is also an important factor with regard to the relationship of this macronutrient with immune system. In this line, it has been highlighted that including proteins of high biological value (those present in eggs, lean meat, fish, and dairy) containing all the essential amino acids may exert an anti-inflammatory effect. In addition, some amino acids, such as arginine and glutamine are well known for their ability to modulate the immune system [7].

Among lipids, the omega-3 fatty acids eicosapentaenoic acid (EPA) and docosahexaenoic acid (DHA) can inactivate enveloped viruses by modulating the optimal host lipid conditions for viral replication. On the other hand, EPA and DHA inhibit cyclooxygenase enzymes (COX) and, thus, may help suppressing prostaglandin (pro-inflammatory) production [3]. Moreover, they are enzymatically converted to pro-resolving mediators (SPMs), such as protectins, resolvins, and maresins, alleviating inflammation [8]. According to these effects, the supplementation with DHA and EPA may be useful to reduce the severity and/or improve the recovery of patients with COVID-19. On the other hand, polar lipids, such as phospholipids, glycolipids or sphingolipids (also present in food sources of omega 3 fatty acids, such as fish and fish oils) have the ability to block platelet-activating factor (PAF) as well as its receptor, exerting anti-inflammatory effects that may be beneficial in COVID-19. Moreover, it has also been described that these lipid species can also down-regulate the enzymes involved in PAF biosynthesis, as well as up-regulate those involved on its degradation [9]. The blockage of platelet activation may also be useful to prevent the thrombotic complications associated to COVID-19 [3].

Carbohydrates and dietary fiber have also been reported to be related to immune system function. As far as carbohydrates is concerned, the consumption of those with higher glycemic indexes (highly processed carbohydrates) can result in mitochondrial overload and subsequent free radical synthesis. Indeed, increased circulating levels of inflammatory cytokines such as C reactive protein (CRP), tumor necrosis factor alpha (TNF-α), and interleukin-6 (IL-6) have been reported with the consumption of these kind of carbohydrates [10]. Due to the aforementioned inflammatory status that usually occurs in respiratory infections such as COVID-19, limiting the consumption of foods rich in these carbohydrates may be advisable.

With regard to fiber, its importance for a correct metabolic functioning has been widely reported. Several studies have revealed that an adequate fiber intake (25–35 g/day) may help reducing both, systemic and gut inflammation. Indeed, the consumption of foods that are source of fiber has been related to lower levels of inflammatory cytokines (CRP, TNF-α, and IL-6), as well as enhanced levels of short chain fatty acids (SCFAs) [7]. In this regard, it has been described that different SCFAs (acetate, propionate, and butyrate) have a direct anti-inflammatory effect by inhibiting the release of pro-inflammatory molecules and by decreasing the expression of nuclear factor ĸB (NF-ĸB). Moreover, SCFAs also play an important role in the maintenance of an adequate gut microbiota by increasing the diversity, as well as enhancing the presence of specific health-associated bacteria [11]. Besides gut microbiota, nasopharyngeal microbiota may also be involved in respiratory infections. Indeed, it has been reported that this kind of infections may result in altered gut microbiota and innate immune system response [12]. Taking into account that COVID-19 has been related to respiratory and gastrointestinal symptoms, it seems plausible that gut microbiota impairment may occur, which in turn can result in an enhanced inflammatory status.

Vitamins A, C, D, E, B6, B12, and folate, iron, magnesium and trace elements including zinc, selenium and copper [13,14] play a pivotal role in disease susceptibility and the maintenance of immune function (Table 1) [15]. Deficiencies and/or inadequate status in these nutrients may negatively affect immune system, resulting in decreased resistance against infections.

The mentioned vitamins and minerals are essential for adaptive immunity as they are involved in cytokine production, lymphocyte differentiation and proliferation, antibody production, and generation of memory cells. Regarding to innate immunity, they also contribute to the maintenance and development of physical barriers and differentiation of innate cells, production, and activity of antimicrobial proteins, phagocytic activities of neutrophils and macrophages, and regulation of the overall inflammatory response.

Significant research has been conducted over decades regarding the usage of vitamin C as a remedy for colds [3]. In the case of COVID-19, there is no evidence so far to recommend its supplementation. In this regard, a clinical trial devoted to analyzing the effect of vitamin C (24 g/day for 7 days) in 140 patients with severe COVID-19 is being carried out in Wuhan (China) (Identifier: NCT04264533) [27].

The relationship between vitamin A and infections has been extensively described. In the case of respiratory infections, vitamin A plays a pivotal role due to its involvement in healthy mucus layer formation, as well as enhancing antigen non-specific immune response [28]. Indeed, histopathological alterations have been described in pulmonary epithelium and parenchyma in subjects with vitamin A deficiency, resulting in impaired respiratory function [29]. Consequently, adequate intakes of this vitamin should be guaranteed in order to avoid further complications in the case of COVID-19.

The association of vitamin D deficiency with respiratory tract infections and lung injury has been widely reported. Indeed, the usage of vitamin D agonists has shown effectiveness ameliorating the aforementioned conditions [30]. Furthermore, previous investigations have demonstrated that high-dose supplementation of vitamin D (250,000–500,000 IU/day) is safe and effective in improving the health status of mechanically ventilated critically ill patients (enhancing the capacity of blood for oxygen transport and increasing hemoglobin levels), which resulted in shorter hospital stays [31,32]. With regard to COVID-19, Ilie et al. have studied the role of vitamin D levels on the prevention of this infection and the mortality induced by this disease [33]. The authors have determined the mean levels of 25(OH)D in subjects from 20 European countries, as well as the number of positive cases and deaths caused by COVID-19. They have found a negative association between the mean levels of vitamin D and the number of COVID-19 cases, as well as with the mortality. They also observed that vitamin D levels are severely low in the aging population, which is the most vulnerable population group for COVID-19.

In the same line, a retrospective study carried out in Switzerland has shown that patients positive for SARS-CoV-2 have lower levels of 25(OH)D than non-infected subjects [34]. However, when patients were stratified by age, the mentioned difference was only significant among patients older than 70 years, who are the subjects in higher risk to suffer clinical complications in COVID-19. Based on the results obtained, the authors proposed to reach a plasma concentration of 30 ng/mL 25(OH)D in this population, in order to reduce the risk in case of serious disease.

Nevertheless, not all the results go in the same direction. Thus, taking into account that black and minority ethnic individuals seem to be more affected by COVID-19, vitamin D deficiency is being studied to explain this fact. In a study conducted by the UK Biobank, 1474 participants were submitted to COVID-19 test, being positive 449 of them. Among the 1474 subjects, 95% were white people (12% of them were vitamin D deficient), 1% were black people (36% of them were vitamin D deficient), 2% were South Asian subjects (57% of them were vitamin D deficient) and the remaining 2% belonged to other ethnic groups (29% of them were vitamin D deficient). Among all the participants, 25(OH)D concentration was lower in those patients who subsequently had confirmed COVID-19 infection. Moreover, 25(OH)D predicted COVID-19 infection when displayed an univariate analysis, but not after it was adjusted for potential confounders. Univariate analysis showed that black and South Asian ethnicities were associated with confirmed COVID-19 infection compared with whites, although adjustment for 25(OH)D serum concentrations showed no significant associations between vitamin D nutritional status, ethnicity, and an increased susceptibility to COVID-19 infection. Authors concluded that the potential role of 25-hydroxyvitamin D concentration to explain susceptibility to COVID-19 infection could not be supported overall or in blacks and minority ethnic populations [35]. Nevertheless, it should be highlighted that after the publication of these results, two letters to the editor showing some weak points of this study have been published [36,37].

As shown in the previous lines, the studies carried out to date have reported controversial results regarding vitamin D levels and the risk to develop COVID-19. Consequently, there is no enough evidence to recommend supplementation of this vitamin in those subjects who do not exhibit deficits. In this regard, meeting the recommended dietary allowances for this vitamin would be the best advice. By contrast, higher doses of vitamin D are advisable for vulnerable subjects in order to avoid deficits, especially in situations of lockdown [3].

Vitamin E has been related to the correct function of the humoral and innate immune functions. Indeed, the ability of vitamin E to scavenge reactive oxygen species (ROS) plays an important role in oxidative stress reduction, exerting anti-inflammatory effects. In addition, vitamin E also protects polyunsaturated fatty acids (PUFAs) and immune cells from oxidation. To date, there is little evidence regarding the use and/or dosage of vitamin E as a prophylactic or therapeutic agent against COVID-19.

Iron is a nutrient with diverse implications in COVID-19. On the one hand, it is well known the importance of iron for the correct functioning of the immune system. However, it is also well established that iron-containing enzymes are essential for the completion of virus replication process, particularly coronaviruses [21]. Thus, it has been pointed out that iron chelation could be an alternative adjuvant strategy to treat COVID-19 patients, via manipulation of key iron regulators (still needs further research) or via venous injection or oral administration of iron chelators. In this sense, previous treatments with iron chelator deferiprone (DFP, Ferriprox^®^) prolonged the survival of patients with acquired immunodeficiency syndrome (AIDS) after human immunodeficiency virus (HIV) infection [38,39]. Evidence also suggests that iron chelators can exhibit antiviral effect on HIV through the elevation of intracellular iron efflux and increasing iron exporter ferroportin expression [40]. Despite to date little is known about iron regulation in COVID-19 patients, it could be deduced from other viral infections that iron chelation might be an alternative beneficial adjuvant in treating COVID-19. However, it is important to point out that there is no empirical research to date, so further investigation is needed.

Selenium plays an essential role in the immune system due to its anti-inflammatory effect. Zhang et al. (2020) have identified a positive association between higher recovery rate from COVID-19 infection and adequate selenium status in 17 cities outside the region of Hubei (China) [20]. This effect is in line with the significant benefits of selenium supplementation demonstrated against other viral infections, including HIV [41,42], hepatitis B linked liver cancer linked [43] or epidemic hemorrhagic fever [44].

Interestingly, the amount of trace elements present in food varies according to the geographical differences of the soil. In this regard, soils in different regions of China have been reported to have the highest and lowest selenium levels in the world. Zhang et al. (2020) have found that infected patients from the areas with high selenium levels were more prone to recover from COVID-19 [20]. Indeed, the cure rate (percentage of COVID-19 patients declared as recovered) was almost threefold higher on the city of Enshi in Hubei Province, which has the highest selenium intake in China, compared to the other cities in Hubei. By contrast, in Heilongjiang Province, where selenium intake is among the lowest in the world, the death rate from COVID-19 was almost fivefold higher than the average of all the other provinces outside of Hubei. These observations suggest that selenium intake seem to be related to the clinical outcome in COVID-19 patients. However, further research is still needed in order to provide more specific advice regarding the adequate intake of this mineral.

With regard to zinc, te Velthuis et al. (2010) demonstrated that increasing Zn^2+^ concentration inhibits the replication of SARS-coronavirus (SARS-CoV) [45]. On the other hand, zinc deficiency is linked with defective cell-mediated immune response, as well as with increased susceptibility for various infections. Indeed, it has been suggested that increased zinc intake may exert beneficial effects on COVID-19 infections by reducing gastrointestinal and lower respiratory symptoms. In addition, it has been suggested that zinc intakes of 30–50 mg/d may exert beneficial effects on RNA viruses [46].

Copper is essential to maintain DNA integrity by preventing oxidative DNA damage. Studies conducted in rodent models with chronic TNF-α-induced lung inflammation proposed that copper supplementation may ameliorate such inflammation [47]. However, no substantial evidence is available in order to recommend copper supplementation against COVID-19.

**Figure 2:**
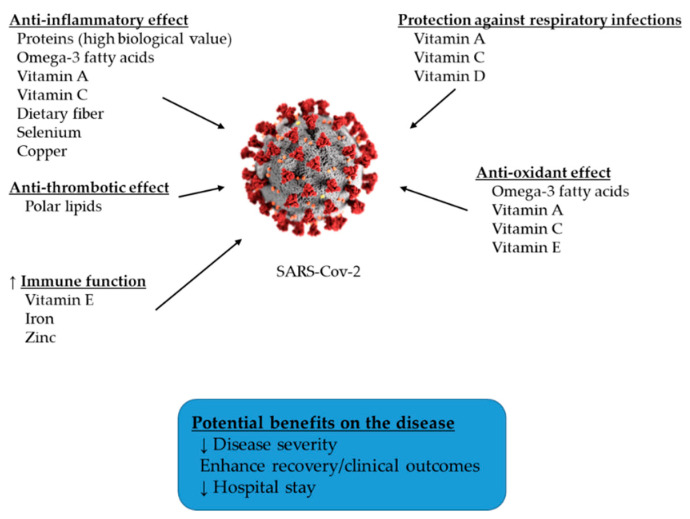
Effects of several nutrients on aspects of COVID-19 infection. ↑: increase, ↓: decrease.

Figure 2 shows the effects of several nutrients on immune system function and other important aspects on COVID-19 infection, such as oxidative stress, inflammation, and thrombosis.

## 3. The Role of Specific Bioactive Compounds Present in the Diet on Immune System Function and COVID-19 Disease

**Figure 3:**
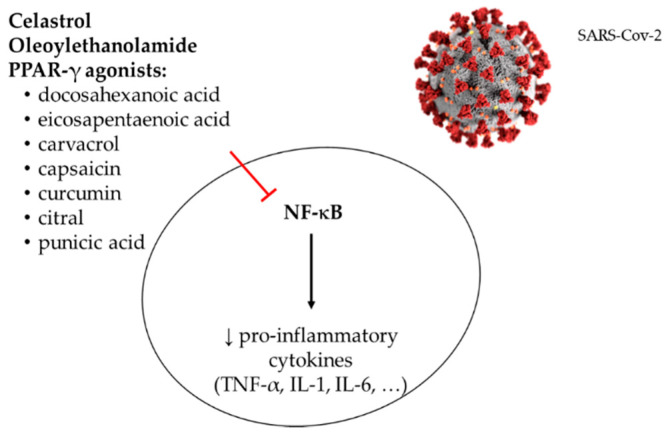
Anti-inflammatory effect of bioactive compounds present in foodstuffs. PPAR: peroxisome proliferator-activated receptor, TNF: tumor necrosis factor, IL: interleukin, ↓: decrease.

Nowadays, there is an urgent need to identify molecules effective to prevent COVID-19 disease and/or to reduce its severity. In this regard, it has been found that several natural bioactive compounds interact with ACE2 receptor, which is the gateway for SARS and SARS-CoV-2, and thus regulates the viral infection. Natural bioactive compounds can also reduce the inflammatory response induced by SARS-CoV-2 infection. Taking into account that a great number of the patients with COVID-19 present “pro-inflammatory cytokine storm”, which drive to a worse prognosis, these molecules could represent a promising target for immunomodulatory therapies. In the following lines, the potential benefits of resveratrol (3,5,4′-trihydroxy-*trans*-stilbene), celastrol, oleoylethanolamide, and natural peroxisome proliferator-activated receptor γ (PPAR-γ) agonists (Figure 3) are described. Nevertheless, it should be pointed out that the vast majority of these results have been obtained in animal models; thus, further studies are still needed to check them in humans.

### 3.1. Resveratrol

Resveratrol is a polyphenol belonging to the group of stilbenes, naturally found in red wine, berries, grapes, nuts, and other foodstuffs. Horne et al. (2020) reviewed the biological interaction between resveratrol and the ACE2 receptor described in four studies that have been published to date [48]. As explained in the Introduction section, SARS-CoV-2 uses the ACE2 receptor to enter the mammalian cells, as well as the transmembrane protease serine 2 (TMPRSS2), involved in the viral spike-(S) protein cleavage, which in turn mediates the fusion of virus and cellular membranes necessary for viral entry (Figure 1). Therefore, due to the role of ACE2 in SARS-CoV-2 infection, it could be thought that nutrients or molecules (such as some bioactive compounds and antihypertensive drugs) that may enhance the expression/synthesis of this enzyme should be avoided in order to reduce the risk of infection. However, it must be noted that ACE2 activation also exerts beneficial effects on the lung injury produced by SARS-CoV-2 infection [3]. Thus, due to the dual role that ACE2 may play in COVID-19, further research is needed in order to better understand whether its activation represents a beneficial or a detrimental effect in the development of the disease.

Tiao et al. (2018) observed, in a study carried out in rats, that resveratrol increases ACE2 protein levels in the liver, at a dose of 50 mg/kg/day [49]. Oliveira Andrade et al. (2014) reported that mice fed a high-fat diet supplemented with resveratrol revealed increased ACE2 gene expression in adipose tissue compared with mice fed the high-fat diet alone [50]. Kim et al. (2018) studied the effect of this natural compound in thoracic aortas of 24-month-old mice, observing an increased expression of ACE2 protein and an enhanced protection against arterial aging [51]. There is also an in vitro study carried out in human aortic smooth muscle cells, in which resveratrol incubation for 24 h up-regulated ACE2 protein [52].

### 3.2. Celastrol

**Figure 4:**
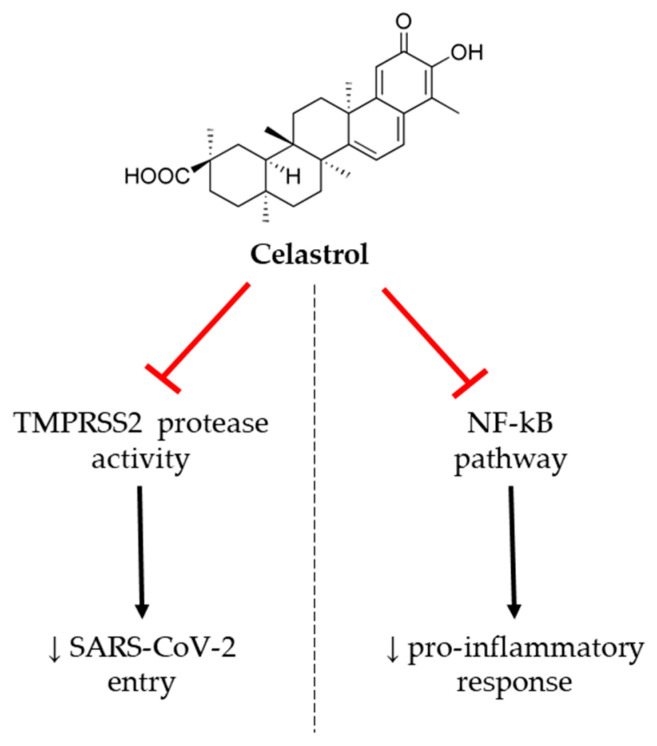
Anti-inflammatory activity of celastrol. NF-κB: nuclear factor *kappa* B, TMPRSS2: transmembrane protease serine 2, ↓: decrease.

Habtemariam et al. (2020) reviewed the effect of celastrol, a pentacyclic triterpenoid, which has shown anti-inflammatory benefits on lung diseases, in animal models, by suppressing NF-ĸB signaling [53] (Figure 4). In the study reported by Shi et al. (2018), this compound improved chronic obstructive pulmonary disease by decreasing interleukin-8 (IL-8), TNF-α, and monocyte chemoattractant protein-1 (MCP1) levels, as well as by increasing enzymatic antioxidant defenses in mice [54]. These results are in good accordance with those previously reported by Wei and Wang (2017), who observed that celastrol reduced these pro-inflammatory cytokines and NF-kB activation in rats, showing lipopolysaccharide (LPS)-induced acute respiratory distress syndrome [55]. More recently, Iwata-Yoshikawa et al. (2019) showed that NF-kB pathway was regulated by TMPRSS2 protein in the airway of a TMPRSS2 knockout murine model, after coronavirus infection [56]. In this sense, the authors suggested that celastrol could be a promising candidate for COVID-19 treatment due to its capacity to inhibit TMPRSS2 protein and, thus, reduce the cleavage of S protein and the subsequent viral entry.

### 3.3. Oleoylethanolamide

It is well known that the activation of immune system receptors called toll like receptors (TLRs) reduces the expression levels of PPARs, thus activating the NF-kB pathway and leading to the release of cytokines IL-6 and interleukin-1β (IL-1β) [57,58]. Ghaffari et al. (2020) suggested that oleoylethanolamide (OEA), derived from oleic acid (omega-9 monounsaturated fatty acid) and synthesized in the gastro-intestinal tract, may activate PPAR-α receptors and thus prevent gene expression of inflammatory cytokines [59]. This is supported by the results observed in a recent clinical trial where OEA decreased serum levels of IL-6 and TNF-α in obese patients [60]. These facts have encouraged the authors to conduct a clinical trial to evaluate this hypothesis on patients infected with COVID-19 in Iran.

### 3.4. Natural PPAR-γ Agonists

In viral infections, pro-inflammatory cytokines (e.g., TNF-α and IL-1) are released by lung-epithelial cells, endothelial cells and immune cells. PPAR-γ, a transcription factor member belonging to the PPAR family, is known as a regulator of inflammatory response. It suppresses the expression of the pro-inflammatory M1 macrophages and activates the proliferation of anti-inflammatory M2 macrophages. Moreover, it acts on the transcription of inflammatory cytokine genes and inhibits the production of cyclooxygenase-2 (COX-2), an enzyme that induces inflammatory response [61].

In this context, natural PPAR-γ agonists present in foodstuffs could also act as anti-inflammatory molecules by inhibiting the expression of pro-inflammatory cytokines. Ciavarella et al. (2020) have published a review where the anti-inflammatory effect of several natural compounds are summarized [62]: (a) EPA and DHA, provided by sea food and fish oil [63], (b) carvacrol, a monoterpenic phenol present in thyme and oregano, two plants of the Mediterranean area [64], (c) capsaicin, contained in hot pepper, one of the most used spices around the world [65], (d) carnosic acid and carnosol, two dipertenoids present in rosemary and sage [66,67], (e) punicic acid, contained in pomegranate seed oil [68], (f) citral, present in lemongrass oil [69], and (g) curcumin [70,71].

The effects of curcumin on SARS-CoV-2 infection have been addressed by several research groups. As early as 2007, Wen et al. determined the quantity of spike proteins in cultures of Vero E6 cells infected with SARS-CoV, and observed that this compound was able to significantly decrease the virus replication [72]. Later on, Ting et al. (2018), using porcine epidemic diarrhea virus (PEDV) as a coronavirus model, showed that curcumin could suppress viral replication by inhibiting the synthesis of negative-strand RNA virus [73]. Recently, Zahedipour et al. (2020) have shown that curcumin may target critical steps of the viral infection by reducing the penetration of the virus and attacking the components necessary for the viral replication cycle [74].

It is important to point out that some patients infected with COVID-19 develop pulmonary fibrosis, which is mediated by the transforming growth factor β (TGF-β) pathway. With regard to this signaling pathway, it has been reported that curcumin induces a reduction in a mouse model of viral-induced acute respiratory distress syndrome [75]. Moreover, curcumin can reduce type I collagen protein and TGF-β mRNA levels in rodent models with fibrosis [76]. Although the anti-fibrotic effects of curcumin have not been tested in a model of COVID-19, similar effects in this case may be hypothesized.

### 3.5. Probiotics

The gut microbiota is in continuous bidirectional interaction with the host, regulating both adaptive and innate immune systems. It is well known that alterations in intestinal microbiota composition and its metabolites influence organs involved in metabolism, such as adipose tissue or liver, producing metabolic inflammation [77]. Nowadays, great attention is paid to the presence of microorganisms in the lung, an organ rich in bacterial colonies. Indeed, a crosstalk between both organs known as “gut-lung axis” has been proposed [78]. Consequently, exogenous factors, such as antibiotics, diet, or exposition to toxics, that alter gut microbiota, could cause lung inflammation through the mentioned axis, thus increasing morbidity of fibrosis or pneumonia, which are clinical outcomes of COVID-19. Taking this into account, it has been recently postulated that probiotics can exert protection against viral infections at three different levels: (a) providing a stronger innate immune response in the gut, (b) decreasing the gut permeability, and (c) regulating the acquired immune response [79].

In a pilot study, fecal samples of patients diagnosed with SARS-CoV-2 infection (confirmed via polymerase chain reaction (PCR) tests) were collected during hospitalization and compared with samples of healthy individuals [80]. COVID-19 patients presented more opportunistic pathogens and a depletion of commensals in the gut, while healthy subjects maintained a higher prevalence of *Eubacterium, Roseburia, Lachnospiraceae Eubacterium, Faecalibacterium prausnitzii, Roseburia*, and *Lachnospiraceae*. Right now, there are three registered clinical trials devoted to studying the effect of probiotics on COVID-19 patients [79]. One of them is a preventive study that evaluates the beneficial effects of *Lactobacillus coryniformis* on the incidence of COVID-19 illness in healthcare workers exposed to SARS-Cov-2 (NCT04366180). Other clinical trial is evaluating the effect of bacteriotherapy in the treatment of patients with acute diarrhea, and in the prevention of intensive cares in COVID-19 patients (NCT04368351). In the third one, the adjuvant use of oxygen-ozone therapy, along with probiotic supplementation, is investigated in patients with COVID-19 (NCT04366089).

Based on these ideas, probiotics could be proposed as potential tools to be included in the nutritional treatment of COVID-19 patients. Thus, Renzo et al. (2020) have suggested the potential therapeutic use of probiotics such as *Lactobacillus rhamnosus* and *Bifidobacterium lactis*, which exhibit anti-inflammatory effects, and prebiotics for restoring the innate and adaptive immunity [81].

## 4. Protein-Energy Malnutrition

When addressing COVID-19 disease, the study of nutritional status is very relevant since it plays an important role on the functionality of immune system, necessary to face the virus infection. Indeed, malnutrition is associated with immune dysfunction and thus it is likely to assume that this condition could make individuals more vulnerable to the viral infection [82,83].

On the other hand, nutritional status can be negatively affected by the SARS-CoV-2 itself, as well as by the applied treatments. Hospitalized patients with COVID-19 tend to present malnutrition at the time of hospitalization. Chronic diseases that are commonly present in patients with COVID-19 (mainly diabetes, chronic obstructive pulmonary disease, renal insufficiency, cardiovascular diseases or dementia), as well as other risk factors such as socio-economic status or frailty, have negative effects on the nutritional status of these patients. In addition, during hospital stay, the prolonged immobilization, mainly in long stays in intensive care units (ICU), leads to muscle mass losses, making the recovery of these subjects harder. Furthermore, the need for assisted breathing during prolonged periods also contributes to the development of sarcopenia and malnutrition [84,85,86,87,88]. This deteriorated nutritional status seems to be involved in the virulence of the virus, and probably in the clinical outcome. In this regard, studies conducted in Italy have demonstrated the importance of maintaining/recovering an adequate nutritional status in the clinical outcomes of the patients [89,90].

Due to fluid administration and rapid wasting of lean tissues, weight and BMI changes do not accurately reflect malnutrition in COVID-19 patients. Thus, the loss of lean body mass is of more concern than that of the BMI. Indeed, loss of muscle and sarcopenia have to be detected, since the larger the muscle mass decrease is, the more severe the malnutrition will be [91].

Malnutrition is probably due to anorexia, nausea, vomiting, and diarrhea (which impair food intake and absorption), hypoalbuminemia, hypermetabolism, and excessive nitrogen loss [92,93]. These effects are clearly associated with the increase in pro-inflammatory cytokines observed in these patients. Moreover, anorexia can also be related to dysgeusia. Lechien et al. conducted a study devoted to analyzing the effect of COVID-19 infection on gustatory disorders [94]. For this purpose, 417 mild-to-moderate COVID-19 patients (164 males and 263 females) with a mean age of 37 years old were recruited from 12 European hospitals. More than 88% of the patients reported gustatory dysfunction, which was characterized by impairment of salty, sweet, bitter, and sour tastes. The gustatory disturbance consisted of reduced/discontinued or inaccurate capability to taste flavors in 79% and 21% of patients, respectively. In these subjects, olfactory and gustatory dysfunctions were positively correlated. In summary, this study identified gustatory dysfunction as symptom of the European COVID-19 infection. In addition, Zayet et al. (2020) carried out a retrospective study in 217 adults in France, where patients were divided in 2 groups: patients infected by COVID-19 confirmed by a positive PCR on nasopharyngeal sample (95 patients) and patients with a negative PCR result on nasopharyngeal sample (122 patients) [95]. In patients positive in COVID-19, 65% had dysgeusia, whereas in patients negative in COVID-19, only 16% had this symptom. This difference in the frequency was statistically significant. The authors concluded that dysgeusia is frequently reported in patients consulting from COVID-19. Other authors also showed similar results [96,97,98,99]. It is still unknown the mechanism by which gustatory dysfunction takes place, but according to Printza et al. (2020), SARS-CoV-2 may enter into tongue taste cells via the ACE-2 receptor, which not only is present in pulmonary tissue but also in taste organs (at least in mice) [100]. Accordingly, the usual function of the sensory cells can be disturbed.

Several studies have shown the results of nutritional status assessment in various cohorts of COVID-19 patients. Li et al. (2020) carried out the first study aimed to evaluate the nutritional status in elderly patients suffering COVID-19 [101]. For that purpose, a cross-sectional study was designed including 182 patients (65 male and 117 female) from a hospital in Wuhan (China), with a mean age of 69 years and positive in COVID-19. The nutritional assessment of the participants was performed by using the Mini Nutritional Assessment (MNA), a tool for institutionalized geriatric patients. MNA takes into account food intake, weight loss, BMI, morbidity, major or acute psychological diseases, and cognitive status. In these patients, MNA mean score was 22.9, which means a risk of malnutrition; in fact, among the patients, 53% were malnourished, 28% at risk of malnutrition and 20% non-malnourished. There were no differences in age, gender, triceps skin-fold thickness, mid-arm circumference, hypertension, or cerebrovascular, cardiovascular, and chronic lung diseases among the three groups. However, there were statistical differences in the incidence of diabetes mellitus, BMI, calf circumference, albumin, hemoglobin, and lymphocyte counts among the three groups, being the highest values in the group without malnutrition and the lowest in malnourished group. Finally, the multivariate regression analysis showed that diabetes, low calf circumference, and low albumin levels were independent risk factors for malnutrition in these subjects. In view of these results, the authors concluded that in Wuhan (China) there was a high prevalence of malnutrition in elderly patients with COVID-19.

Liu et al. (2020) aimed to evaluate the nutritional risk, as well as their associated clinical outcomes, in older patients with COVID-19. To do so, a retrospective study was carried out in 141 patients (68 males and 73 females), older than 65 years (average age 72 years), 77 of them with hypertension or/and diabetes, showing different status of COVID-19 (46 common COVID-19, 73 severe COVID-19 and 22 extremely severe COVID-19), treated in a hospital from Wuhan (China) [102]. Participants were classified as normal group (no nutritional risk) or as nutritional risk group, according to the criteria of each nutritional risk tool used. The nutritional screening risk methods used by researchers were Nutrition Risk Screening 2002 (NRS 2002), Malnutrition Universal Screening Tool (MUST), MNA short form (MNA-sf), and Nutrition Risk Index (NRI). NRS 2002 predicts patients who would benefit from nutritional treatment in hospital settings and takes into account the BMI, body weight loss, food intake and the severity of the disease. MUST is useful for identifying the necessity of nutritional treatment and for assessing the risk of malnutrition based on different factors like BMI, unintentional weight loss and the presence of acute disease factors to detect disease related malnutrition. NRI is a screening tool that predicts patient’s malnutrition status, based on serum albumin, as well as, on current and usual body weight.

Depending on the used nutritional screening risk method, the assignation to each tool to normal or nutritional risk groups was as follows: 85% of the patients with NRS 2002, 41% with MUST, 77% with MNA-sf and 60% with NRI were assigned to the risk group. These results show that, independently of the used nutritional assessment method, a high percentage of the older patients suffering COVID-19 showed malnutrition.

As far as anthropometric measurements and clinical outcomes are concerned, statistical analysis did not show differences between the normal group and the nutritional risk group. By contrast, with MUST tool BMI was lower in nutritional risk group than in normal group. When using NRS 2002, the fever duration was measured, which resulted to be longer in the nutritional risk group when compared to the control group. Serum albumin and total protein levels were also higher in the control group than in the nutritional risk group, by using all the nutritional risk tools, with the exception of MUST. 

When data were adjusted for age, sex, presence of co-morbidities and BMI, NRS 2002, MNA-sf, and NRI methods demonstrated that patients from the nutritional risk group showed longer hospital stay, lower appetite, worse severity of the disease, and greater weight loss than the patients in the normal group. By using NRS 2002 and NRI methods, patients with nutritional risk also showed higher hospital expenses than normal patients.

## 5. Recommendations for Nutritional Treatment

Although nowadays, the knowledge regarding the nutritional support during hospital stay of COVID-19 patients is still limited, nutritional therapy appears as first-line treatment and should be implemented into standard practice [7,103,104]. In spite of that, due to the priority assigned to urgent pathologies like respiratory issues, the nutritional status of patients has been relegated to a second place. In fact, almost half of the hospitalized polymorbid and 23–60% of patients in acute care are not correctly nourished [105,106]. Other facts that have also exacerbated this situation are that medical teams cannot invest enough time to grant an optimal nourishment due to work overload, staff shortages (healthcare personnel have suffered a high infection rate) or insufficient availability of personal protective equipment. Additionally, a restriction to family visits has been applied in most affected countries, removing a support in nourishment. Moreover, in most hospitals clinical teams and structures have been reorganized resulting in provisional limitations of dietetic support.

The general recommendation for COVID-19 patients is to follow healthy diets to maintain a correct immune function [3]. Optimal intake of all nutrients, mainly those that play crucial roles in immune system, should be assured through a diverse and well-balanced diet. However, current data suggest that there is a prevalent micronutrient and omega-3 fatty acid deficiency in several population groups [107,108,109]. On the other hand, in the review reported by Calder et al. (2020) [16], based on several meta-analysis [110,111,112,113,114,115,116,117,118], the authors state that in order to promote the optimum functioning of the immune system and to reduce the risk and consequences of infections, the intakes for some micronutrients may exceed the recommended dietary allowances since infections and other stressors can reduce micronutrient status. Thus, supplements may help restoring their normal blood levels [16,119,120,121]. With regard to supplementation, it is important to advise the general public to always consult a medical doctor prior consuming such products, as they can interact with other nutrients, drugs, and medical treatments; indeed, they can turn into toxic elements causing several disorders and aggravating certain conditions.

### 5.1. Nutritional Therapy in Non-Critically Ill Hospitalized COVID-19 Patients

Cintoni et al. (2020) described the nutritional management strategy adopted in Fondazione Policlinico A. Gemelli-IRCCS (Rome, Italy), which is considered a reference center for COVID-19 in the region [122]. Due to the mean age of their patients (65 years), their nutritional status is usually not optimal. Moreover, the low palatability of hospital meals makes the energy and protein intake low, which can exacerbate the bad nutritional status of the patients. In this scenario, the orally feedable patients of COVID-19 receive a personalized meal provision, which is completed with oral nutritional supplements in order to meet the energy and protein requirements. In the case of patients who are unable to eat, enteral/parenteral nutritional formulas, rich in protein and poor in glucose, are provided. In this regard, previous studies conducted in China have demonstrated that the inclusion of nutritional support in the treatment of symptomatic COVID-19 patients is of major importance. The conclusion of these studies is that the design of specific nutritional strategies are needed in hospitals [123].

Caccialanza et al., (2020) have also proposed an empirical protocol to promptly and pragmatically implement nutritional care in hospitalized non-ICU COVID-19 patients [89]. The authors recognized that their protocol might be debatable, since it may not be in total agreement with the current guidelines on clinical nutrition [124]. However, they do believe that nutritional care in hospitalized non-ICU COVID-19 patients might be overlooked despite being potentially effective in preventing the consequences of malnutrition and beneficial to clinical outcomes in this patient population [89]. The authors have based their protocol on the observation of the symptoms shown by the patients at hospital admission (severe inflammation, anorexia leading to a major reduction of food intake, and respiratory failure). Since a significant number of patients reported severe eating difficulties, they decided to start with an oral supplementation with whey proteins (20 g/d) and intravenous multivitamin, multi-mineral, and trace element solutions, in order to match the recommended dietary allowances upon admission. Whey proteins were selected based on their high digestibility, anabolic and antioxidant properties [125], immunomodulatory [126], and potential antiviral activities [127]. Further, since specific vitamin and micronutrient deficits have been reported to be harmful during viral infections, including COVID-19 patients [7], the authors justify the micronutrient intravenous administration to meet recommended dietary allowances. The most debatable aspect of their protocol might be the election of parenteral nutrition over enteral nutrition. This election is due to the respiratory difficulties of COVID-19 patients. Certainly, enteral nutrition requires the presence of a nasogastric tube, which may compromise the effectiveness of non-invasive ventilation or continuous positive airway pressure [89]. Further, enteral feeding implies an increased risk of aspiration of the nutritional solution [128]. Indeed, this protocol shows debatable limitations, but taking into account that underfeeding occurs in patients hospitalized for COVID-19, a rapid intervention in non-critically ill patients can improve their prognosis, therefore reducing the pressure on the limitation in ICU availability detected in hospitals worldwide [129].

Once the acute phase of the pneumonia is over, COVID-19 patients need specific rehabilitation, not only to recover the respiratory capacity but also to improve disability and quality of life. Brugliera et al. (2020) describe their experience working in the COVID-19 rehabilitation unit in the Hospital of San Raffaele Scientific Unit (Milan, Italy) with 50 COVID-19 patients [6]. According to the authors, the majority (90%) of the patients showed some degree of dysphagia, mainly due to previous orotracheal intubation, making necessary the use of diets with modified consistency or nasogastric feeding, and highlighting the importance of nutritional support, as well as of swallowing training to improve the recovery of these patients. Due to the direct relationship between nutritional status and the risk of suffering from different illnesses (among them the COVID-19), this parameter was studied by means of the MUST, and the results showed that 45% of the studied population were in high risk of malnutrition, while 26% had moderate risk.

Based on the knowledge acquired by the authors while working with patients who were recovering from COVID-19, a 3-step nutritional protocol has been designed. The first step of this protocol would be focused on the nutritional assessment and malnutrition screening of the patient. For this purpose, different anthropometric parameters, as well as the body composition of the patients are studied. Further, the weight loss is monitored and a hematochemical analysis of blood parameters (including, among others, blood count, total protein, ferritin, blood sugar and markers of liver function) carried out. Finally, the swallowing capacity of the patients (in order to determine whether a specific diet is needed) is evaluated and their intake assessment monitored. Once this first step is completed, a second step devoted to setting the nutritional treatment of the patient takes place. In this regard, the energy and macronutrient requirements of the patient are assessed, using the meal management computerized system of the hospital. In this scenario, energy requirements are calculated using predictive equations, which are adapted to the nutritional status of the patients (clinical status, physical activity, or stress). As far as macronutrients are concerned, a protein intake of >1 g/kg/day (up to 1.5 g/kg/day) is guaranteed, as well as carbohydrate and lipid requirements stablished based on the non-protein energy (30:70 in patients with no respiratory insufficiency and 50:50 in patients with respiratory insufficiency). In addition, maintaining an adequate hydration of the patient is another aspect of the intervention that must be taken into account. In this regard, the clinical history of the patient (heart or renal failure, vomiting or diarrhea) must be analyzed. Moreover, additional supportive therapy (adequate vitamin and oligoelement intake, essential and branched amino acids and probiotics) is also provided. Similarly, nutritional advice that can be useful for the patient (while in the hospital as well as once they are discharged) is provided. As far as the third step is concerned, this is based in continuous monitoring of the patient by a multidisciplinary team over time, which allows modifying the treatment according to the status of the patient.

Using this protocol, the MUST improved in 5 of the 32 patients (out of the 50 patients that were included in the rehabilitation unit) that stayed at least for 10 days in the COVID-19 rehabilitation unit. Moreover, 14 of these patients improved their BMI while in 15 patients this parameter remained stable. Based on these observations, the authors conclude that nutritional status plays a pivotal role in the clinical outcomes of patients recovering from COVID-19, and that nutritional support along with rehabilitation may improve the chances of recovery in patients of COVID-19.

### 5.2. Nutritional Therapy in Critically Ill Hospitalized COVID-19 Patients

Some COVID-19 patients experience severe respiratory symptoms and/or multi-organ failure, being very ill at hospital admission and thus needing specialized support [99,123]. Indeed, acute respiratory complications, requiring prolonged ICU stays, are a major cause of morbidity and mortality in COVID-19 patients. Most of these patients rapidly progress from cough to dyspnea, and then to respiratory failure requiring mechanical ventilation. Consequently, the timing of nutritional intervention appears to be critical [130]. Therefore, nutritionists should choose the most appropriate way to recover the subject’s health.

As with any other critically ill patients, nutritional management is an integral component of good supportive care. The European Society for Clinical Nutrition and Metabolism (ESPEN) has recently published some guidelines for the nutritional management of patients with SARS-CoV-2 infection. In these guidelines specific recommendations are included for patients hospitalized in ICUs, among them early enteral nutrition (when possible), use of agents that promote gastric emptying, initiation of peripheral nutrition in situations in which enteral nutrition is not possible/tolerated and use of enteral nutrition after extubation when oral feeding is not tolerated. According to these ESPEN guidelines, enteral nutrition is preferred for patients in the ICU who receive mechanical ventilation [91]. However, the specific needs of patients with COVID-19 may require the adoption of prone ventilation or neuromuscular blockade, and consequently enteral nutrition implementation in daily practice could be difficult [128]. Further, a delay of enteral nutrition can be mandatory when life-threatening hypoxemia occurs [91].

On the other hand, the changes induced by the disease itself in the gastrointestinal tract of the patients, along with the elevated sedation required for these patients, makes difficult to provide adequate nutritional support. Bearing this in mind, Arkin et al. (2020) provided information regarding several of the considerations that have to be taken into account when nutrition is provided to critically ill patients of COVID-19 [131]. One such consideration is related to the gastrointestinal hypomotility that is commonly found in these patients, which in turn results in enteral feeding intolerance. Indeed, this situation seems not to be derived from the high-doses of sedatives and opioids that are needed to facilitate mechanical ventilation, since other patients under similar conditions do not present feeding intolerance. Thus, the authors suggest that the enteral feeding intolerance is related to the infection produced by the SARS-CoV-2. Moreover, despite several pharmacological agents devoted to promoting gastrointestinal motility are provided to COVID-19 patients at the time of ICU admission, their intestinal motility remains impaired. Additionally, even in the cases in which gastrointestinal motility is maintained/recovered (by studying gastric residuals and stool output), impaired nutrient absorption has been described. In the case of the patients in which gastric residuals are >500 mL, the use of a post-pyloric feeding tube is recommended by the ESPEN guidelines. However, this approach involves technical challenges in the placement as well as increased exposure of the staff to viral infection and obvious difficulties when proning of the patient is required. Besides the gastrointestinal features of critically ill COVID-19 patients, severe lung injury is also common in these subjects. This condition makes necessary strategies devoted to avoiding aspirations. In this regard, the authors suggest holding tube feeding 1-h prior proning as an effective approach to avoid aspirations. Indeed, the authors state that due to the challenges related to aspiration in prone patients, continuous tube feeding is maintained (instead of using bolus tube feeding).

As far as diet is concerned, the ESPEN guidelines recommend hypocaloric nutrition in the first week of ICU stay. However, since the patients normally spend days/weeks sick at their homes before being admitted in a hospital, the risk of developing malnutrition increases. Moreover, the life-threatening features that are common on these patients at the time of ICU admission produces further delay in enteral nutrition, increasing even more the risk of malnutrition. Due to these facts, critically ill COVID-19 patients may have poor nutritional status, which in turn may increase the severity of the infection. In this regard, the authors point towards the necessity of a more aggressive early total parenteral nutritional support in these patients.

Taking all that into account, and considering that COVID-19 patients need more energy than normal, the nutritional management of the critically ill patient in the long-term should avoid underfeeding or overfeeding. It is recommended to supply around 20 or 30 kcal/kg body weight/day for polymorbid patients aged >65 years or for severely underweight polymorbid patients respectively [124,132]. These values should be adjusted with regard to nutritional status, disease status, and tolerance [133]. It is important to take into account that in severely underweight patients, the target of 30 kcal/kg body weight/day should be slowly achieved (in the case of artificial nutrition, target speed must be reached in 3–4 days) due to the high risk of refeeding syndrome of this population [104,132].

Protein needs are higher in the critically ill patient due to the protein catabolism driven by inflammatory mediators [132]. It is estimated as 1 g protein/kg body weight/day in older people [133], or 1.3 g protein/kg body weight/day with increased supply of branched chain amino acids (up to 50% in polymorbid medical inpatients), in order to prevent muscle loss and to strength respiratory muscles [132]. Other authors increased these recommendations to 1.5 g protein/kg body weight/day [89]). These amounts should be individually adjusted with regard to nutritional status, disease status, and tolerance [124].

The needs of fat and carbohydrate have to be adapted to the energy requirements, considering an energy ratio from fat and carbohydrates between 30:70% for individuals with no respiratory deficiency and to 50:50% for ventilated patients [104]. Fat requirement in the critically ill patient is around 1.5 g/kg body weight/day, giving priority to the usage of medium and long chain fatty acids. As far as carbohydrates requirement is concerned, a supply of 2 g/kg body weight/day, not exceeding 150 g/day, must be considered. This is due to their high CO2 production rate that should be limited in case of respiratory failure [132].

With regard to micronutrients in ICU patients, mineral and vitamin supply with routine supplements must be considered depending on the nutritional therapy used. Administration of doses higher than recommendations of vitamin D can be used because of their positive effects [132]. Certainly, it has been reported that high doses of Vitamin D in infected patients may improve immunologic recovery during antiretroviral treatment, reduce levels of inflammation and immune activation, and increase immunity against pathogens [89]. In turn, vitamin C supplementation significantly reduces mortality in critically ill patients [134].

In the case of enteral nutrition, attention must be paid to the needs of specific nutrients to be included in the formula. Thus, enteral diets containing EPA, gamma-linolenic acid, and antioxidant agents may offer a clinical benefit in oxygenation and days of ventilation in patients with acute respiratory distress syndrome [91]. Further, specific lipid emulsions could offer additional benefits over corticosteroid and anti-IL-6 drugs in the modulation of the inflammatory response. However, COVID-19 patients’ response to specific enteral diets remains to be determined [130].

## 6. Future Research

In the present review, we provide data about key aspects in nutritional management of COVID-19 patients, based on the current knowledge. Nevertheless, due to the novelty of the disease, information regarding the effects of some nutrients is still scarce.

Regarding the role of specific nutrients in COVID-19 disease, very often the nutritional advice is based on their effects in infections caused by other viruses with symptoms similar to those caused by SARS-CoV-2. On the other hand, in some cases the studies reported are ecological studies, which are the first step in the investigation of a possible relationship between a disease and a determined exposure. Their great advance is that they are carried out very quickly, practically without cost and with information that is usually available. However, their main limitations are that they cannot determine if there is an association between an exposure and disease at the individual level, and their inability to control for potentially confounding variables. Consequently, further research based on analytical instead of descriptive studies, such as randomized controlled clinical trials, are warranted.

Regarding the effects of bioactive compounds present in foodstuffs on COVID19, the vast majority of the results have been obtained in preclinical studies. One advantage of these studies is that animals can be directly exposed to the virus under treatments with various bioactive compounds, as a first step to know if some of these substances could reduce or even prevent infection with the SARS-CoV-2 virus. Moreover, they allow the researchers to determine the mechanism involved. In this field of research, studies addressed in humans are needed to confirm the beneficial effects observed in animals.

Another important aspect is to focus the research on specific age groups, paying special attention to the elderly, since it is known that the evolution of the disease depends largely on age. In the same way, it would be important to carry out separate studies in men and women since it appears that men are more negatively affected by this disease. All this will contribute to making a personalized nutritional approach, and therefore more successful.

## Figures and Tables

**Table 1:** Recommended intakes of certain nutrients with key roles in disease susceptibility and the maintenance of an adequate immune function.

Nutrient	Immune Function	Recommendation		References
		Healthy Individuals	Diseased/Infected Patients	
Vitamin C	Maintenance of functional and structural integrity of mucosal cells in innate barriers Normal functioning of T cells Antimicrobial, anti-inflammatory and antioxidant effects Antibody production Reduction of respiratory tract and lung infection risk	200 mg/day	1–2 g/day	[16,17]
Vitamin D	Maintenance of functional and structural integrity of mucosal cells in innate barriers Normal functioning of T cells Antimicrobial, anti-inflammatory and antioxidant effects Antibody production and antigen responses Reduction of respiratory tract and lung infection risk Alleviation of the inflammatory response	2000 IU/day (50 µg/day)	10,000 IU during few weeks, followed by 5000 IU (until 25–hydroxyvitamin D concentrations rise above 40–60 ng/mL (equivalent to 100–150 nmol/L))	[16,18,19]
Vitamin E	Maintenance of functional and structural integrity of mucosal cells in innate barriers Differentiation, and functioning of innate immune cells Anti-inflammatory and antioxidant effects Antibody production and antigen responses Reduction of respiratory tract and lung infection risk Support of T cell-mediated immunity	15 mg/day (RDA)	200 IU/day	[16]
Selenium	Differentiation, and functioning, of innate immune cells Normal functioning of T cells Antibody production Antimicrobial, anti-inflammatory and antioxidant effects	50 µg/day	Up to 200 µg/day	[17,20]
Zinc	Maintenance of functional and structural integrity of mucosal cells in innate barriers. Differentiation, and functioning, of innate immune cells. Antimicrobial, anti-inflammatory and antioxidant effects. Antibody production and antigen response. Support of lymphocyte and cytokine functions, and innate immunity overall. Inhibits the activity and replication of coronavirus (SARS-CoV which caused an outbreak in 2002)	Men: 8 mg/day Women: 11 mg/day (RDA)	Zinc lozenges: over 75 mg/day administered within 24 h (divided into 6–8 doses, each separated by 2–3 h when awake) Zinc gluconate: 13.3 mg/day within 3 days (at least)	[16,17,18,21,22,23]
Iron	Maintenance of functional and structural integrity of mucosal cells in innate barriers Differentiation, and functioning, of innate immune cells Normal functioning of T cells. Antimicrobial, anti-inflammatory and antioxidant effects	Men: 8 mg/day Women age 19–50: 18 mg/day Women age > 51: 8 mg/day (RDA)	Ferrous iron salts (ferrous sulfate and ferrous gluconate): 60 mg Fe/day (taken with food to avoid gastric discomfort)	[17,24]
Omega-3 fatty acids (EPA + DHA)	Conversion to specialized pro-resolving mediators (SPMs) such as, protectins, resolvins and maresins to relieve the inflammation and enhance lung injury	250–300 mg/day of EPA + DHA	1500–3000 mg/day EPA + DHA	[16,25]
Multivitamin supplements including vitamins (A, B6, B12, C, D, E and folate) and trace elements (Zn, Fe, Se, Mg and Cu)	Support of the cells and tissues of the immune system overall Maintenance and development of in innate barriers Growth and differentiation of innate cells Antibody production and generation of memory cells Production and activity of antimicrobial proteins Phagocytic activities of neutrophils and macrophages	Supplying nutrient requirements according to the 100% RDA for age and gender This is in addition to a well-balanced diet		[16,25,26]

DHA: docosahexaenoic acid, EPA: eicosapentaenoic acid, RDA: Recommended Dietary Allowances.
